# WHO-definition of health must be enforced by national law: a debate

**DOI:** 10.1186/1472-6939-14-24

**Published:** 2013-06-19

**Authors:** Marion Habersack, Gero Luschin

**Affiliations:** 1 Office of the Vice-Rector for Teaching & Studies, Medical University of Graz, Auenbruggerplatz 2, Graz, 8036, Austria; 2 Womens Health Association, Quellengasse 36, Graz, 8010, Austria

**Keywords:** WHO, Health, Right to health, Democratic process

## Abstract

**Background:**

On its establishment, the World Health Organization (WHO) defined health as a fundamental human right deserving legal protection. Subsequently, the *Ottawa Charter* reaffirmed health as a fundamental right, and emphasized health promotion as the most appropriate response to global health issues. Here we suggest that the WHO definition of health as more than simply the absence of illness is not normative, and therefore requires standardization. To date such standardization unfortunately is lacking.

**Discussion:**

National legislatures must actively ensure fair access to health care, both nationally and internationally, and also must reduce social inequality. To achieve this requires practical action, not statements of intentions, commitments or targets. Protecting fundamental rights to health care can be a fruitful focus for legislatures. Legislative action can build an objective legal framework for health care law, and guide its interpretation and application. Additionally, it is important to ensure the law is appropriate, useful and sustainable.

**Summary:**

Action is needed to protect the fundamental right to health care. Legislators should appropriately incorporate the WHO recommendations regarding this right into national law. Additionally, professional experts should help interpret and codify concepts of health and join the interdisciplinary discussion of a variable health standard.

## Background

The foundation of the World Health Organization (WHO) as a special organisation of the UN not only determined the responsibilities of the organisation itself, but also defined health as a fundamental human right that deserves legal protection [1]. Some years later, the *Ottawa Charter* responded to global health issues partly through health promotion and thus reaffirmed health as a fundamental right [2]. Here we suggest that the WHO definition of health as more than just the absence of illness is not normative, and therefore requires standardization that thus far is lacking.

“Health is the state of complete physical, mental, and social well-being and not merely the absence of disease and infirmity” [1]. The recommendation of the WHO that health be seen as more than just the absence of disease, and as a fundamental human right, was formulated more than half a century ago. This view has since been confirmed by numerous subsequent WHO documents, but remains inadequately implemented at the level of national law and standardised codes of practice despite WHO requests [3]. This results in unequal access to health care, and ultimately social inequality, by excluding most potential users from the development of standards and policy.

## Discussion

### Demystification

Current thinking about the significance of the WHO and its recommendations approaches deliberate mystification. Discussions in the health sciences regularly invoke the WHO and its declared commitment to the basic human right of health—made in passing and without any reference to law—as a binding foundation for decisions and actions, and a component of national laws. Although this interpretation only partially expresses the truth, it indicates an intradisciplinary view (initially merely theoretical with respect to the health sciences, but ultimately having practical implications) of international organisations and fundamental human and civil rights. The WHO’s significance lies not in its ability to enforce health standards at the levels of national and international law through implementing health schemes but rather in the political sphere. The WHO is relevant in the way it expresses prevailing (or more importantly, not yet prevailing) convictions regarding the importance of health, including its legal regulation, and the evolution of a process-based conception of health. The WHO’s recommendations, including its declaration of the fundamental human right to health, merely express abstract universal ideals, and provide no foundation for a fundamental human right to health. Human rights first designate moral rights, from which moral claims can be derived, and which have a purely moral legal basis. This gives rise to a claim these rights are universally valid, independent of sociocultural, historical, political, religious or other kinds of constellations and cultures. To define human rights on this level implies that their validity is independent of the mechanisms of legal enforcement. The application or enforcement of the law, or hypothetical legal claims based on the declaration of a fundamental human right to health, requires that such a right be written into law at the national level, and that legal mechanisms be established by which individuals can assert his or her rights. Responsibility for implementing this change lies with individual countries, and particularly with experts in their public health care systems, otherwise the idea will remain an idea and no more.

To ensure fair access to health care, at both the national and international levels, and to reduce social inequality, national legislatures must expand their intervention in healthcare beyond declarations of intent, stipulation of national targets or stated commitments [4].

Intervention in this area by national legislatures is limited for the following reasons:

(1) The right to health as defined by the WHO is merely a basis for argument and a point of departure, and lacks the status ascribed to the core concepts of human rights.

(2) Sovereignty and property, which represent the needs of the privileged, obstruct the universal validity, feasibility of implementation and acceptance of even a minimalist conception of a fundamental right to health.

(3) The formulation of macrosocial ideals by international institutions cannot create structures that maximize health. Maximization of health depends on access to health care institutions, and regardless of moral components, any universal right to health must be primarily legal. Such a right requires concrete and binding standards and statutory implementation. The right to health asserted by the WHO therefore must be transposed into ‘hard law’ and thus made directly applicable and mandatory.

### More than nothing but still not enough

This transposition has not been adequately effected by individual governments. Neither experts working in the health care system, for whom a fundamental national right to health is highly significant to the legal concretisation of health care provisions, nor persons who wish or are compelled to exploit the health care system, can appeal to ‘hard law’.

Increasingly complex requirements are reducing latitude for action, thus preventing objectively appropriate, requirement-oriented and sustainable planning or supply of health related services. The results range from inadequate response through to ignorance of specific problems. The consequent unfair treatment excludes certain groups from participation in health services and leads to a lack of social acceptance of those services among the excluded. What is not available to all cannot be the object of democratic legitimacy, and is unsuitable for formalization in national law. The conclusion that a specific group exclusively influences and benefits from standards and standardisation related to health may seem obsolete but is not trivial [5,6]. This is because fundamental rights are generally subject to the same principle as human rights and only create the conditions for a right to health when they express real social values. An exclusively instrumental conception of a right to health not only cannot provide genuine content or yield a practical approach to the exercise of that right, but also enjoys no absolute priority over other conceptions of value. Such a right can only be realized in a society that determines and guarantees appropriate fundamental structures, such as the principles of universal freedom and equality.

In the political sphere, a fundamental right to health can only be achieved by drafting a standard that establishes limits on that right. This requires the support of the health sciences, clear differentiation, and repeated and ongoing scrutiny. Interdisciplinary cooperation is thus particularly important in matters of health. Determining the meanings of health, equitable treatment and realizing equality are problems to be solved cooperatively by both medicine and the legal sciences.

## Summary

### Future prospects

Action is clearly needed. Besides the call for appropriate incorporation of the WHO recommendations in national law, professional experts in particular are being called on to help interpret and codify as yet ‘unsaturated’ concepts of health and contribute to the necessary interdisciplinary discussion of a variable standard. In doing this experts must consider the following:

(1) Existing forms of standardisation as well as the future provision of health services depend on social acceptance. Without popular participation in the creation, application and interpretation of a right to health, the actualization of this right will never extend beyond institutions reactively providing basic health care.

(2) The formulation of standards does not necessarily equate with social acceptance. Standards inevitably reflect the interests of their drafters, and generate norms that transcend the regulated area. Particularly in the case of health, standards describe not only the values of a society but also its actual distribution of resources.

(3) Health is a political issue, particularly when defined as more than the mere absence of disease. The fundamental legal structure of many states is heavily oriented to safeguarding basic economic rights (property rights, freedom to earn etc.). Change in these structures to prioritize health will encounter resistance [7].

(4) The common practice has been to define health standards in terms of sickness rather than health. This results in a negative definition of health as the absence of disease, which fails to reflect the reality that health is a continuum, with emotional or social components and embedded in an extensive social context.

## Competing interests

The authors declare no competing interests.

## Authors’ contributions

Both authors contributed equally to the manuscript.

## Pre-publication history

The pre-publication history for this paper can be accessed here:

http://www.biomedcentral.com/1472-6939/14/24/prepub
